# Extracorporeal Support Prognostication—Time to Move the Goal Posts?

**DOI:** 10.3390/membranes11070537

**Published:** 2021-07-15

**Authors:** Neel Shah, Ahmed S. Said

**Affiliations:** Division of Pediatric Critical Care, Department of Pediatrics, School of Medicine, Washington University in St. Louis, St. Louis, MO 63130, USA; said_a@wustl.edu

**Keywords:** ECMO, predictive scores, decision making

## Abstract

Advances in extracorporeal membrane oxygenation (ECMO) technology are associated with expanded indications, increased utilization and improved outcome. There is growing interest in developing ECMO prognostication scores to aid in bedside decision making. To date, the majority of available scores have been limited to mostly registry-based data and with mortality as the main outcome of interest. There continues to be a gap in clinically applicable decision support tools to aid in the timing of ECMO cannulation to improve patients’ long-term outcomes. We present a brief review of the commonly available adult and pediatric ECMO prognostication tools, their limitations, and future directions.

## 1. Introduction

Extracorporeal Membrane Oxygenation (ECMO) has seen its use exponentially increase over the last two decades [1]. New compact devices, improvements in anti-coagulation strategies and monitoring, and improvements in cannulation techniques have continued to fuel the growth within the field, and more centers are now offering ECMO support [1]. 

As the technology has advanced, the field has seen the criteria for ECMO deployment continue to expand, with progressively more centers pushing the boundaries and offering ECMO for previously contraindicated disease processes and even in out-of-hospital settings. ECMO mortality has slowly improved with recent trials demonstrating mortality in the 30–50% range, compared to over 40–70% in similar patients not receiving ECMO [2,3,4,5]. 

Despite these advances, the fundamental question of prognostication persists—who is a good candidate for ECMO support? 

This question has never been more pressing than during the recent COVID-19 pandemic. As resources are stretched thin, offering resource-intensive therapies approaches a zero-sum situation in which difficult decisions must be made [6,7,8,9,10]. To date, the common approaches have focused on identifying factors in either patient characteristics or complications, mostly prior to ECMO initiation, to develop prognostication scores. The developed scores have mostly relied on registry-based data, especially for neonatal and pediatric scores, and with mortality as the main outcome. We review the current prognostication tools available to clinicians, their limitations, and the future directions of the field. 

## 2. Current ECMO Prognostication Tools

Over the last two decades, several groups have put together prediction scores with Area Under Receiver Operator Curve (AUC), ranging from 0.65 to 0.89 depending on the group and indication evaluated. 

### 2.1. Neonatal Respiratory Failure

Two current scores exist to predict ECMO outcomes in neonatal respiratory failure, both derived from the Extracorporeal Life Support Organization (ELSO) registry. Both the Pittsburgh Index of Pre-ECMO Risk (PIPER) score [11] and Neonatal Risk Estimate Score for Children Using Extracorporeal Respiratory Support (Neo-RESCUERS) [12] rely on a combination of demographic, laboratory, and treatment data prior to ECMO cannulation to predict survival. 

The PIPER score demonstrated a 21% increase in mortality for each PIPER quartile. The mortality data was also separated by presence or absence of CDH. They further presented a combined model including ECMO duration and presence of complication categories as independent variables and showed an improved AUC of 0.79.

Neo-RESCUERS analyzed neonates over overlapping periods of the registry to create a development and validation dataset. They showed that patients in their lowest decile had an observed mortality of 7% compared to a predicted mortality of 4.4%, while the highest decile had an observed mortality of 65.6% compared to a predicted mortality of 67.5%. 

Both scores present reasonable AUCs but neither have been externally validated (Table 1). ECMO cannulation technique has also changed over time with more neonatal veno-venous (VV) ECMO cannulations occurring than from the time this registry data was collected.

### 2.2. Neonatal Congenital Diaphragmatic Hernia

There is one neonatal congenital diaphragmatic hernia (CDH) ECMO prediction tool that is designed to be scored either with only variables from prior to cannulation or with the addition of variables after cannulation [13]. The highest risk group within the pre-ECMO score had a mortality of 75% compared to the mortality of the highest risk group as defined by the on-ECMO score of 86%. As expected, the addition of on-ECMO variables results in an improvement in the accuracy of the model (Table 2). These findings illustrated the importance of ECMO complications when predicting mortality.

### 2.3. Pediatric Respiratory Failure

Two scores exist for pediatric respiratory failure (Table 3): the Pediatric Risk Estimation Score for Children Using Extracorporeal Respiratory Support (PED-Rescuers) score [14] and the newer Pediatric Pulmonary Rescue with Extracorporeal Membrane Oxygenation Prediction (P-Prep) score [15]. Both scores were developed utilizing data from the ELSO registry with an overlap in the data collection period but with a slight difference in the patient cohort age group. The PED-Rescuers score was developed and then validated against a dataset also from the ELSO registry, while the P-Prep score underwent external validation against another large pediatric database, the Pediatric Health Information System (PHIS). Both scores rely on pre-ECMO data regarding demographics including diagnosis, as well as laboratory and treatment variables. Since they were published, the incidence of pediatric respiratory ECMO has continued to increase, and the ELSO registry has transitioned to ICD-10 coding from ICD-9, which may provide more granular diagnosis data. Additionally, the ELSO registry underwent extensive revision and standardization of all data fields in 2018 to provide a more standardized approach across centers and to allow for more reliable multicenter data analysis [16].

### 2.4. Adult Respiratory Failure

Several Adult Respiratory Failure scores exist (Table 4), with the earliest being ECMOnet developed on data from the 2009 H1N1 Pandemic [17]. This score was limited to a single country cohort entered into an international research collaborative, ECMOnet [18]. They analyzed 60 patients, finding an overall survival rate of 68%. Predictors included hospital length of stay prior to ECMO, bilirubin, creatinine, hematocrit, and mean arterial pressure. These variables were split into categorical values and translated into scores. The external test set revealed AUC of 0.69 with an accuracy of 62%, sensitivity of 51%, and specificity of 62%. 

Since that time, several other scores have been developed. Utilizing data from three French intensive care units (ICU), the PRedicting dEath for SEvere ARDS on VV-ECMO (PRESERVE) score was developed, including 140 patients with refractory acute respiratory distress syndrome (ARDS) and eight pre-ECMO parameters as predictors [19]. Infectious diseases were the leading cause of ARDS with 26% being infected with influenza H1N1. They found that the pre ECMO P:F ratio was not associated with survival, and due to trials around this time showing improved mortality with early prone positioning, the authors forced it into their model and found it was independently associated with lower mortality. In addition to aiming to identify factors associated with long-term mortality for ICU survivors at 6-months post-discharge with 80% of survivors participating, they looked at health-related quality of life (HRQOL) and reported emotional (34%), anxiety (25%), depression or post-traumatic stress (PTSD) symptoms (16%). They further compared HRQL outcomes in their population compared to controls, as well as other ECMO and ARDS trials. 

The Respiratory Extracorporeal Membrane Oxygenation Survival Prediction (RESP) score was developed with ELSO registry data and then externally validated on single center data used to develop the PRESERVE score [20]. It was further validated in several other independent studies [21,22]. Survival within the registry data was 57%; several diagnosis groups were associated with mortality, and acute non-pulmonary infections and central nervous system dysfunction were also significant predictors. Included variables were then scored, and the final score was split into five risk classes with risk class I demonstrating 92% survival vs risk class V with 18% survival. Prone positioning was not reported within the registry and thus was not a predictor. The RESP score also performed better in the external database compared to classical ICU severity scores such as simplified acute physiology score II (SAPS II) and sequential organ failure assessment (SOFA).

Both RESP and PRESERVE scores have also been compared in separate independent databases, where they were found to have similar accuracy in predicting both in-hospital and 6-months survival [23,24]. Of note—four variables overlap between the scores including age, immunocompromised status, mechanical ventilation time prior to ECMO, and peak inspiratory pressure (PIP) or plateau pressure. 

The Roch score focused on identifying factors associated with mortality in patients referred to a single center for acute respiratory distress syndrome (ARDS) and then requiring ECMO support [25]. They looked at 85 consecutive ARDS patients equipped with ECMO by a mobile team, with the median time to cannulation 3 h after contact from the referring center. The variables were assigned scores with the 0–2 score group having 40% mortality and the 3–4 score having 93% mortality. The strength of the Roch score is its development in a referral center setting. 

While several other scores have been developed on either multi or single center data and then been externally validated, in general they have achieved good accuracy in predicting mortality with only limited variables. These include the Veno-venous (VV) ECMO mortality score [26] and PREdiction of Survival on ECMO Therapy-Score (PRESET) score [27]. The PRESET score was derived with 108 ARDS patients on VV-ECMO who were retrospectively analyzed with the EMCOnet, RESP, PRESERVE and Roch score. Within their dataset, only the ECMOnet and RESP score accurately determined survival, and subsequently the PRESET score was created including extrapulmonary predictors such as mean arterial pressure, lactate, pH, platelets, and hospital days pre-ECMO. The scores were then split into three risk classes with risk class I having 26% mortality vs risk class III with 93% mortality. 

A recent single center external validation also looked at these scores (PRESERVE, RESP, VV-ECMO mortality, and SOFA scores). The authors found slightly lower AUCs than previously reported between 0.64–0.73 [21]. 

### 2.5. Adult Cardiac Failure

Three large adult prognostic scores for survival for cardiac veno-arterial (VA) ECMO exist (Table 5). The SAVE score is based off the ELSO registry data [28]. It was designed to predict survival from refractory cardiogenic shock. Registry data showed a 42% survival, the score was then validated on 161 Australian patients. The score was then split into risk classes from I to IV, with survival in class I at 75% vs. class IV at 18%. They further compared these risk categories across the most prevalent diagnosis groups, with myocarditis and heart and lung transplants having the highest survival. The external validation AUC was quite high (0.90), but it was done on a single highly protocolized center with higher survival and difference in diagnosis distribution. This center also participated in the ELSO registry so there may be some overlap between these cohorts. 

The prEdictioN of Cardiogenic shock OUtcome foR AMI (Acute Myocardial Infarction) patients salvaGed by VA-ECMO (ENCOURAGE) score was based off multicenter data of 138 patients and also followed up on HRQOL data [29]. It was developed over 138 patients from two centers and was unique in that it was the first specific risk score for AMI-cardiogenic shock. They demonstrated 47% survival and had seven pre-ECMO predictors. Their HRQOL data was compared among score classes with 6-month survival ranging from 7 to 80%. They also reported anxiety (34%), depression (20%), and PTSD (5%) among their survivors. 

Conversely, the Predict VA-ECMO score is based off variables for patients already on ECMO, relying on very few variables at different time intervals with good accuracy [30]. They were able to develop a dynamic score using only three points of care biomarkers, and it outperformed the SAVE score within their database. A significant strength was the evaluation of extracorporeal cardiopulmonary resuscitation (eCPR) patients as well as cardiogenic shock patients. 

## 3. Limitations in Current Prognostic Scores

The majority of prediction models aim to predict the same outcome—mortality. Aside from CDH on-ECMO score and Predict VA-ECMO, all scores take input variables from pre-ECMO cannulation to help predict mortality. As one would expect looking at the variables of interest, a patient’s severity of illness and degree of end organ dysfunction prior to ECMO initiation often plays a large role in their predicted mortality. Unfortunately, there are limited trials to help establish when a patient would best benefit from cannulation, as both too early and too late cannulation could be associated with adverse outcomes. In other words, the current models do not provide clinicians with decision support tools for timely institution of ECMO support, before end organ deterioration develops that has been shown to correlate with worse in-hospital and long-term mortality. 

While several adult scores have been designed without registry data or are externally validated, many pediatric scores are not externally validated. External validation is often crucial to ensure a prediction score is applicable to varying patient cohorts with differences in demographics, therapeutics, and even diagnoses. Recent studies have shown even externally validated adult scores performed similarly and with lower accuracy than initially reported [23,24]. Recent literature has been published on how some of these scores performed retrospectively during the COVID-19 pandemic [2]. Recent evidence from the COVID-19 pandemic has also shown that the timing of ECMO initiation influences patient outcomes [32]. 

A common factor is that a large portion of predictive variables only point to existing end organ dysfunction prior to cannulation but provide scant evidence on modifiable risk factors either in the pre or on ECMO phases. They also mostly predict only short-term mortality, but provide limited information on long-term neurologic or functional outcomes. 

## 4. Future Directions

As described in a recent joint paper regarding the development and reporting of prediction models from respiratory, sleep, and critical care journals—a significant limitation of prediction models is the inclusion of casual relationships between variables [33]. Variables such as pre-ECMO cardiac arrest or SOFA score are independently related to mortality—with or without ECMO cannulation. Furthermore, the joint paper discusses the limitations of categorizing continuous variables and the subsequent loss of information. For example, a model that treats hypotension, mean arterial pressure (MAP) <60 mmHg, as a binary variable, may treat MAPs of 39 and 59 mmHg the same, as well as the folly of relying only on AUC curves without considering the importance of precision and recall, especially when datasets are unbalanced [34]. As survival improves overall, especially for conditions such as CDH, it is increasingly likely that there will be significantly unbalanced classes (higher survival). Both these limitations impact the vast majority of current ECMO prediction scores.

Further, while current prediction scores can inform clinical decisions regarding population level statistics of who will survive ECMO, they offer little in the way of input on how to improve these outcomes. Most rely on demographics and pre-cannulation markers of severity of illness or pre-ECMO cardiac arrest [35,36]. Research has also focused on factors that relate to continued multiorgan failure such as need for renal replacement therapy [23,37], continued need for vasoactive support, or markers of hemolysis or disseminated intravascular coagulation [38,39]. 

Little has been offered in terms of ECMO predictive scores that alert clinicians of actionable variables that may impact outcomes. Recent adult literature has shown in a large ELSO database that a significant relative decrease in PaCO_2_ in respiratory ECMO patients was associated with poor neurologic outcomes [40]. Pediatric data has shown the importance of PaCO_2_ and its interaction with blood pressure on cerebral autoregulation in a single center [41]. Studies in pediatric patients have also shown the impact of hyperoxia on neurologic outcomes [42,43]. Other literature has focused on the impact of cannulation modality and mortality [44,45]. 

Factors such as relative changes in PaCO_2_, MAP, cannulation Site, and degree of hyperoxia are often modifiable by experienced clinical staff. Predictive scores with a focus on a change in variables over time and their impact on mortality are sorely needed to aid not only in the decision to place a patient on ECMO, but to predict and impact outcomes following cannulation. 

These challenges also remain ever present during the current COVID-19 pandemic, with the RESP & PRESERVE score having AUCs of 0.60 and 0.55 in a multicenter COVID-19 database [2]. The complexity of the COVID-19 patients in that they may require longer durations of mechanical ventilation, may have more significant ARDS and overall increased treatment complexity is not well reflected in either of these scores. We propose new COVID-19-specific scores should be developed that can include these further nuances. We further propose these prediction scores should be under continuous development and revision, which will improve their ability to serve as true clinical decision support tools.

## 5. Changing the Outcome

Deployment of ECMO has become more widespread with nearly a 10-fold increase over the last two decades. Recent trials have also documented the improvement in mortality outcomes provided by ECMO support. As with other interventions that have become more widespread in critical care, the focus shifts from a binary outcome of mortality to more nuanced patient-centered outcomes. 

Neurologic injury is one of the most common complications of ECMO, with neurologic complications such as intracranial hemorrhage associated with upwards of 40% of deaths. Children are similarly affected with survival falling to 36–38% following acute neurologic injury. In children in particular, long term follow up has also demonstrated that survivors often have significant impairment—12% report severe motor impairment, 10–50% with cognitive delays, 16–46% with behavioral abnormalities, and 31–53% of survivors having quality of life scores at least one standard deviation below the mean. Likewise, a standardized 1-year follow up clinic after respiratory ECMO demonstrated neurodevelopmental problems in 30% of the 98 patients [46]. Prior papers have focused on the role of cannulation sites, PaCO_2_ change, and hyperoxia amongst others, for their roles in neurologic injury. Unfortunately, neurologic outcome has not been the focus of prediction models, likely due to heterogenous conditions of patients pre-ECMO, the role of their pre-ECMO illness, and difficulty in collecting and analyzing long term data. Despite these challenges, the next generation of prediction models may benefit from focus on neurologic outcomes rather than survival and ICU discharge, as previously mentioned.

## 6. Conclusions

We present a review of current ECMO prediction models in neonatal, pediatric, and adult ECMO patients developed over the last decade, as well as a challenge for the next generation of models to focus on accuracy as well as precision, clinically modifiable outcomes, and outcomes aside from mortality such as long-term neurologic and functional status. 

## Figures and Tables

**Table 1:** Prognostication scores for neonatal respiratory failure.

Score	Variables	Year	Patient Cohort	AUC (95% CI)	External Validation
**PIPER** [11]	**Prior to ECMO Demographics** Apgar at 5 min <7 Birth weight <3 kg Age >10 days CDH **Vitals** MAP <49 mm Hg **Laboratory Data** pO_2_ <34 mm Hg **Treatment Data** Patient not on iNO	2016	**Source** ELSO registry **Date** 2000–2010 **Age** <30 days old **# of Patients** 5455 on VA ECMO	0.74 (0.72–0.77)	No
**Neo-RESCUERS** [12]	**Prior to ECMO Demographics** Birth Weight Gestational Age Age Gender Primary Diagnosis Comorbidity Renal Failure **Laboratory Data** pH PaO_2_/FiO_2_ **Treatment Data** iNO	2016	**Source** ELSO registry **Date** 2008–2013 **Age** <28 days **# of Patients** 4592 patients	0.77 (0.75–0.80)	No

AUC = area under receiver operator curve, ECMO = extracorporeal membrane oxygenation, # = number, CDH = congenital diaphragmatic hernia, MAP = mean arterial pressure, PaO_2_ = partial pressure of oxygen, iNO = inhaled nitric oxide, VA = veno-arterial, ELSO = Extracorporeal Life Support Organization.

**Table 2:** Prognostication scores for neonatal congenital diaphragmatic hernia.

Score	Variables	Year	Patient Cohort	AUC (95% CI)	External Validation
**CDH** **Pre-ECMO** [13]	**Prior to ECMO Demographics** Prior diaphragmatic hernia repair Critical congenital heart disease Perinatal infection Weight APGARs Side of hernia Pre ECMO-Arrest **Laboratory Data** pH **Treatment Data** Ventilator settings	2018	**Source** ELSO registry **Date** 2000–2015 **# of Patients** 4374 Neonates with CDH as primary diagnosis	0.65 (0.62–0.68)	No
**CDH** **On-ECMO** [13]	**Prior to ECMO + On-ECMO** **Treatment Data** ECMO settings (pump type) ECMO associated complications (hemorrhage, severe neurologic complication, elevated creatinine, dialysis, tamponade, CPR, sepsis)	2018		0.73 (0.71–0.76)	No

AUC = area under receiver operator curve, ECMO = extracorporeal membrane oxygenation, # = number, CDH = congenital diaphragmatic hernia, CPR = cardiopulmonary resuscitation, ELSO = Extracorporeal Life Support Organization.

**Table 3:** Prognostication scores for pediatric respiratory failure.

Score	Variables	Year	Patient Cohort	AUC (95% CI)	External Validation
**PED-RESCUERS** [14]	**Prior to ECMO Demographics** Comorbidities Primary diagnosis of Asthma, Bronchiolitis or Pertussis **Laboratory Data** pH PaCO_2_ **Treatment Data** Ventilator settings Duration of admission and MV prior to ECMO Milrinone	2016	**Source** ELSO registry **Date** 2009–2014 **Age** 29 days to 18 years **# of Patients** 2458 on ECMO for respiratory indications	0.63 (0.60–0.65)	No
**P-PREP** [15]	**Prior to ECMO Demographics** Gender Age >10 years Year of ECMO support Primary pulmonary diagnosis Comorbidities **Laboratory Data** PF ratio pH **Treatment Data** VV vs VA Mechanical ventilation >14 days HFOV iNO Neuromuscular blockade	2017	**Source** ELSO registry **Date** 2001–2013 **Age** >7 days to <18 years **# of Patients** 4352 patients needing ECMO for respiratory failure	0.69 (0.67–0.71)	Yes (PHIS dataset—0.66 (0.63–0.69))

AUC = area under receiver operator curve, ECMO = extracorporeal membrane oxygenation, # = number, PHIS = pediatric Health Information System, MV = mechanical ventilation, CPR = cardiopulmonary resuscitation, ELSO = Extracorporeal Life Support Organization, VV = veno-venous, VA = veno-arterial, HFOV = high frequency oscillatory ventilation, iNO = inhaled nitric oxide.

**Table 4:** Prognostication scores for adult respiratory failure.

Score	Variables	Year	Patient Cohort	AUC (95% CI)	External Validation
**ECMONet [17]**	**Prior to ECMO Demographics** PreECMO hospital length of stay **Vitals** Mean Arterial pressure **Laboratory Data** Bilirubin Creatinine Hematocrit	2013	**Source** Prospective multicenter **Date** 2009 H1N1 Pandemic **# of Patients** 60 H1N1 influenza A patients with respiratory distress	0.86 (0.75–0.96)	Yes—0.69 (0.56–0.83)
**PRESERVE [19]**	**Prior to ECMO Demographics** Age BMI Immunocompromised SOFA > 13 **Treatment Data** MV > 6 days No prone positioning prior to ECMO PEEP < 10 cm H_2_O Plateau Pressure > 30 cm H_2_O	2013	**Source** Multicenter (3 French ICUs) **Date** 2008–2012 **# of Patients** 140 ARDS patients	0.89 (0.83–0.94)	Yes (0.68 (0.62–0.75) and 0.75 (0.57–0.92)) *
**RESP [20]**	**Prior to ECMO Demographics** Age Immunocompromised status Mechanical ventilation prior to initiation of ECMO Acute Respiratory Diagnosis CNS dysfunction Acute associated nonpulmonary infection Cardiac Arrest prior to ECMO **Laboratory Data** PaCO_2_ **Treatment Data** Neuromuscular blockade prior to ECMO iNO HCO_3_ Peak inspiratory pressure	2013	**Source** ELSO registry **Date** 2000–2012 **# of Patients** 2355 Adult patients with Severe acute respiratory failure	0.74 (0.72–0.76)	Yes (0.92 (0.89–0.97) and 0.81 (0.67–0.95)) *
**Roch Score [25]**	**Prior to ECMO Demographics** SOFA Age Influenza Pneumonia	2014	**Source** Single Center **Date** 2009–2013 **# of Patients** 85 patients with ARDS	0.80 (0.71–0.89)	Yes (0.56 (0.45–0.68)) **
**VV [26]**	**Prior to ECMO Demographics** Immunocompromised SOFA score **Treatment Data** Days of MV	2016	**Source** Single center **Date** 2007–2015 **# of Patients** 116 patients on VV ECMO for ARDS	0.76 (0.67–0.85)	No
**PRESET [27]**	**Prior to ECMO Demographics** Hospital days pre ECMO **Vitals** Mean arterial pressure **Laboratory Data** Lactate pH Platelet	2017	**Source** Single Center **Date** 2010–2015 **# of Patients** 108 patients with Severe ARDS treated with VV ECMO	0.85 (0.76–0.93)	Yes (0.70 (0.56–0.84))

AUC = area under receiver operator curve, ECMO = extracorporeal membrane oxygenation, # = number, ELSO = Extracorporeal Life Support Organization, VV = veno-venous, VA = veno-arterial, CNS = central nervous system, iNO = inhaled nitric oxide, BMI = body mass index, SOFA = Sequential Organ Failure Assessment, ICU = intensive care unit, ARDS = acute respiratory distress syndrome. * These findings were validated externally in separate databases not included in the original study ** Validated in the same database as used for PRESET Score.

**Table 5:** Prognostication scores for adult cardiac failure.

Score	Variables	Year	Patient Cohort	AUC (95% CI)	External Validation
**SAVE** [28]	**Prior to ECMO Demographics** Cardiogenic shock Age Weight Pre-ECMO organ failure Chronic Renal failure Pre-ECMO cardiac arrest **Vitals** DBP before ECMO >40 mmHg PP before ECMO <20 mmHg **Laboratory Data** HCO3 < 15 mmol/L **Treatment Data** Duration of intubation prior to initiation of ECMO Peak inspiratory pressure <20 cmH_2_O	2015	**Source** ELSO registry **Date** 2003–2013 **# of Patients** 3846 patients with refractory cardiogenic shock treated with VA ECMO	0.68 (0.64–0.71)	Yes—0.90 (0.85–0.95) 0.77 (0.69–0.86) * [31] & 0.69 (CI unavailable) **
**Encourage** [29]	**Prior to ECMO Demographics** Age Sex BMI > 25 GCS < 6 **Laboratory Data** Creatinine Lactate Prothrombin activity	2016	**Source** Multicenter **Date** 2008–2013 **# of Patients** 138 ECMO treated AMI patients	0.84 (0.77–0.91)	No
**PREDICT VA-ECMO** [30]	**On ECMO Laboratory Data** pH Lactate HCO_3_	2018	**Source** Single Center **Date** 2010–2015 **# of Patients** 205 VA-ECMO	0.82 (0.76–0.88)—6 h score 0.84 (0.78–0.90)—12 h score	Yes—0.72 (0.65–0.78) (6 h) & 0.74 (0.65–0.82) (12 h)

AUC = area under receiver operator curve, ECMO = extracorporeal membrane oxygenation, # = number, ELSO = Extracorporeal Life Support Organization, VA = veno-arterial, DBP = diastolic blood pressure, PP = pulse pressure, AMI = acute myocardial infarction. * These findings were validated externally in separate databases not included in the original study ** Validated in the same database as used for PREDICT VA-ECMO Score.

## Data Availability

Not applicable.
